# Cool-season annual pastures with clovers to supplement wintering beef cows nursing calves

**DOI:** 10.1186/2049-1891-3-25

**Published:** 2012-07-24

**Authors:** Stacey A Gunter, Whitney A Whitworth, T Gregory Montgomery, Paul A Beck

**Affiliations:** 1 United States Department of Agriculture, Agricultural Research Service, Southern Plains Range Research Station, 2000 18th Street, Woodward, OK, 73801-5415, USA; 2 University of Arkansas at Monticello, Monticello, Arkansas, USA; 3 Division of Agriculture, Southeast Research & Extension Center, University of Arkansas, Monticello, Arkansas, USA; 4 Division of Agriculture, Southwest Research & Extension Center, University of Arkansas, Hope, Arkansas, USA

**Keywords:** Annual ryegrass, Beef cows, Clovers, Nitrogen, Pasture

## Abstract

In December of 3 years, 87 beef cows with nursing calves (594 ± 9.8 kg; calving season, September to November) at side were stratified by body condition score, body weight, cow age, and calf gender and divided randomly into 6 groups assigned to 1 of 6 cool-season annual pastures (0.45 ha/cow) that had been interseeded into a dormant common bermudagrass (*Cynodon dactylon* [L.] Pers.)/bahiagrass (*Paspalum notatum* Flugge) sod. Pastures contained 1 of the following 3 seeding mixtures (2 pastures/mixture): 1) wheat (*Triticum aestivum* L.) and ryegrass (*Lolium multiflorum* Lam., **WRG**), 2) wheat and ryegrass plus red clover (*Trifolium pretense* L., **WRR**), or 3) wheat and ryegrass plus white (*Trifolium repens* L.) and crimson clovers (*Trifolium incarnatum* L., **WRW**). All groups had *ad libitum* access to grass hay (12% crude protein; 58% total digestible nutrients). The second week in December, cow estrous cycles were synchronized and artificially inseminated. In late December, a bull was placed with each group for 60-d. Data were analyzed with an analysis of variance using a mixed model containing treatment as the fixed effect and year as the random effect. Body weight and condition scores did not differ (*P* ≥ 0.27) among cows between February and June. Calf birth weights or average daily gain did not differ (*P* ≥ 0.17) among treatments; however, calves grazing pastures with clovers did tend (*P* = 0.06) to weigh more than calves grazing grass only. Weaning weight per cow exposed to a bull was greater (*P* = 0.02) for WRR and WRW than WRG. Cows grazing winter-annual pastures containing clovers tended to wean more calf body weight per cow exposed to a bull than cows grazing the grass only pastures.

## Background

Complementary forage systems based on warm-season perennial grasses and cool-season annual grasses have proven successful for cow/calf production in providing supplemental nutrients and decreasing hay requirements during the winter [1-5]; common advantages noted in these reports are extension of the grazing season and decreased days and quantities of hay feeding required. For example, Gunter et al. [5] reported that interseeding wheat (*Triticum aestivum* L.) and annual ryegrass (*Lolium multiflorum* Lam.) into a common bermudagrass (*Cynodon dactylon* [L.] Pers.) pasture in southern Arkansas completely eliminated the need for grain-based supplementation and decreased the amount of hay required per cow.

A negative issue associated with grass only systems is the need for significant amounts of chemical fertilizer containing nitrogen, such as urea. Nitrogen fertilizer inputs represent a large part of the total feed cost in forage-based livestock systems. Further, nitrogen fertilizers are a major source of nitrous oxide emissions in the feed production for herbivores and more efficient use of fertilizers is an important tool to mitigate nitrous oxide losses [6]. In an Australian experiment, nitrogen loss from total denitrification were 116% less from unfertilized pasture of clover and perennial ryegrass (*Lolium perenne* L.) mixtures compared with all perennial ryegrass pastures fertilized annually with 200 kg of nitrogen/ha from urea [7]. An experiment in northern Florida evaluated the use of wheat or rye (*Secale cereale* L.) with crimson (*Trifolium incarnatum* L.) and arrowleaf (*Trifolium vesiculosum* Savi) clovers as a supplement to Argentine bahiagrass (*Paspalum notatum* Flugge) hay and discovered that winter-annual pasture grazing could decrease hay intake by as much as 30% compared to bahiagrass hay, plus a grain-based supplement [3].

These forage systems have been successful but they increase the cattle enterprise’s need for nitrogen fertilizers. To address this issue, we evaluated the use of *Trifolium* species in cool-season pasture mixtures overseeded in to warm-season pastures to replace the need for fertilizer nitrogen, using the fixation of atmospheric nitrogen by associated *Rhizobium* bacteria.

## Materials and methods

All animal procedures in this experiment at the Southeast Research and Extension Center, Monticello, Arkansas (33° 35’ N, 91° 48’ W) were conducted in accordance with the recommendations of Consortium [8]. Each year 2001 to 2003, during the last week of September through the first of December, 87 cross-bred beef cows (body weight = 552 ± 9.4 kg), of mostly Beefmaster breeding, were allowed to calve in a 5-ha pasture and fed a bermudagrass/bahiagrass hay that averaged 12% crude protein and 58% total digestible nutrients (dry matter basis). Before penning in the 5-ha calving pasture, cows were treated for internal and external parasites with an ivermetin, vaccinated with a 7-way *Clostridial* antigen, and vaccinated against infectious bovine rhinotracheitis, bovine viral diarrhea, parainfluenza-3, bovine respiratory syncytial virus plus 5 strains of Leptospirosis. The morning after calving, calves were weighed, eartagged with an individual number, and male calves were castrated and implanted with zeranol (Ralgro; Schering-Phough Animal Health, Kenilworth, NJ, USA).

In the first week of December, cows were weighed and body condition scores (1 to 9 scale) were recorded [9]. Cows were sorted into 6 groups by body condition score and weight, cow age, and calf gender and assigned to 6 dormant common bermudagrass/bahiagrass pastures (2 pastures/treatment) that had been interseeded to 1 of 3 cool-season grass and(or) clover combinations: 1) pastures were wheat and annual ryegrass (**WRG**), 2) the same grasses as WRG plus red clover (*Trifolium pretense* L., **WRR**), or 3) the same grasses as in WRG plus crimson clover and white clover (*Trifolium repens* L., **WRW**). Groups had *ad libitum* access to the same cutting of bermudagrass/bahiagrass hay as described above.

Cool-season forages were interseeded into the six 4.0- to 6.9-ha common bermudagrass/bahiagrass pastures during the first week of October using a no-till drill (Model 750, John Deere, Inc.; Des Moines, IL, USA). Before planting, standing herbage mass was removed from the area by continuously stocking with cattle until the standing herbage mass was visually estimated to be < 5 cm. After planting, forage was allowed to grow until December so forage was not limiting through January and February, when plant production was less than cattle demand. Seeding rates for the grasses were 101 kg/ha of wheat (variety not specified), 22 kg/ha of ‘Marshall’ ryegrass; clover seeding rates were 8 kg/ha of ‘Cherokee’ red clover, 11 kg/ha of ‘Tibbee’ crimson clover, and (or) 5 kg/ha of ‘Oseola’ white clover. Clovers were inoculated with the appropriate *Rhizobium* bacteria and reseeded annually. In the fall of 2000, pastures had lime applied in amounts sufficient to raise the soil pH to approximately 7.0 [10]. Pastures were annually fertilized with phosphorus and potassium 2 weeks after planting based on soil test [10] plus 55 kg of nitrogen/ha. In late-January, mid-March, and late-June, pastures with no clovers were fertilized with an additional 55 kg of nitrogen/ha on each date using urea. Pastures with clovers received no additional nitrogen fertilizer beyond the initial application that occurred 2 weeks after planting.

During the first week of December, an estrous synchronization protocol was employed, which included vaginal insertion of an implant drug release (1.38 g of progesterone; Eazi-Breed™ CIDR, Pfizer Animal Health, Madison, NJ, USA) for 7 days, with half of the cows receiving an injection of gonadotropin-releasing hormone (100 μg, i.m.) and the remaining receiving estradiol cypionate (2.0 mg i.m.). Prostaglandin F2α (25 mg, i.m.) was injected at CIDR removal on day 7 and an injection of estradiol cypionate (0.5 mg, i.m.) was given 24 to 30 hours after CIDR removal. Cows were inseminated approximately 12 hours after observed standing estrus. These two different estrous synchronization protocols were reported in Whitworth et al. [11]. In this report, conception rates with artificial insemination did not differ (*P* ≥ 0.59) between gonadotropin-releasing hormone or estradiol cypionate and did not (*P* ≥ 0.64) interact with forage system, hence estrous synchronizations protocols were not further considered in the statistical models [11]. Approximately 2 weeks after cows were artificially inseminated, 1 of 6 Angus bulls that had passed a breeding soundness examination was assigned to each of the 6 groups of cows for 60 days.

Cows had *ad libitum* access to a self-fed commercial mineral mixture limited with salt that contained at least 15.0% Ca, 5.0% P, and 5.0% Mg plus 0.13% Cu, 0.30% Zn, and 0.0026% Se. Each year, cows and calves were weighed and body condition score of the cows was recorded again during mid-January, mid-February, late-March, early-May, and early-June. Cows were checked for pregnancy by rectal palpation at weighing in June. Hay intake was not measured in this experiment; research from this location [4] has shown that forage mixtures represented in this experiment were not associated with differences (*P* > 0.20) in hay dry matter intake.

The experiment was analyzed using PROC MIXED (SAS Institute, Inc., Cary, NC, USA) as a completely randomized block (year) design with the effect of treatment (fixed effect) and the covariates of cow age and calving date and the random effect included pasture(year x treatment). Least-square means were separated using the following contrasts: 1) WRG versus WRR and WRW, and 2) WRR versus WRW [12].

## Results and discussion

January through June, body weight did not differ (*P* ≥ 0.27) between cows grazing WRG and the cows grazing WRR and WRW (Table 1). Further, the body weight of cows grazing WRR in February, April, May, and June did not differ (*P* ≥ 0.35) from cows grazing WRW. In January, after the cows had been grazing the winter-annual pastures for approximately 3 to 4 weeks, however, the body weight of cows grazing WRR was greater (*P =* 0.05) than cows grazing WRW; also, this trend (*P* = 0.08) in cow body weight was noted during mid-March. This tendency for greater cow body weight in the early winter for cows grazing WRR probably resulted from red clover seeming to be more productive in the fall and winter, while crimson and white clover were more productive in the late winter and spring. Though species composition of the winter-annual pastures were not measured using a quantitative technology, visual evaluations of the pastures by the research technician during the 3-year experiment resulted in estimates that the pastures with red clover displayed a 20% to 25% canopy cover in mid-winter, while the white and crimson clover pastures only displayed a 10% to 15% canopy cover. Further, during April and May, the red clover diminished to approximately 10% to 15% canopy cover where the crimson and white clovers mixture increased to approximately 40% to 45% canopy cover. In June, white clover was the only remaining *Trifolium* genera occurring in significant amounts in the pastures at a rate of 10% to 15% canopy cover. Cow body condition score did not differ (*P* ≥ 0.34) within any month during the experiment and cows maintained body condition score sufficient to remain reproductively active during the entire year [9,13].

**Table 1:** Body weight, body condition score, body condition score at calving, conception rates and post-partum interval by mature beef cows fed bermudagrass/bahiagrass hay supplemented by grazing on wheat/ryegrass pasture or wheat/ryegrass plus clovers over a 3-year period

	**Treatments**^a^				***P*-value**^b^	
**Item**	**WRG**	**WRR**	**WRW**	**SE**	**WRG versus Clover**	**WRR versus WRW**
Cow body weight, kg						
January	596	608	579	9.8	0.83	0.05
February	596	602	589	11.1	0.95	0.40
March	575	594	565	11.0	0.73	0.08
April	573	577	560	12.7	0.77	0.35
May	599	582	584	11.2	0.27	0.93
June (weaning)	607	594	593	12.6	0.34	0.97
Body condition score ^c^						
January	5.8	5.9	5.9	0.06	0.53	0.76
February	5.9	5.8	5.9	0.09	0.72	0.34
March	5.9	5.8	5.8	0.16	0.71	0.93
April	5.9	5.7	5.9	0.15	0.71	0.55
May	6.0	5.8	5.9	0.14	0.34	0.63
June (weaning)	5.9	5.8	5.8	0.13	0.47	0.86
Conception rate,%	73	83	85	4.9	0.07	0.79
Post-partum interval, d	84	84	98	7.5	0.46	0.19

^a^ WRG = winter-annual pasture composed of wheat and ryegrass; WRR = winter-annual pasture composed of wheat and ryegrass plus red clover, and WRW = winter-annual pasture composed of wheat and ryegrass plus white and crimson clover.

^b^ Contrasts: WRG vs Clover = WRG versus the average of WRR and WRW.

^c^ Body condition score range 1 to 9; 1 = emaciated, 9 = obese [9].

Research at this same location documented the nutritive value of grasses collected from pastures planted to a wheat and ryegrass mixture, similar to the one we used in our experiment, over a 3-year period [14]. These researchers reported [14] that the crude protein concentrations and *in vitro* digestibility (dry matter basis) averaged 15.4 ± 1.3% and 59.0 ± 9.1% in January, 20.6 ± 1.9% and 78.3 ± 2.5% in March, and 17.9 ± 6.6% and 74.3 ±3.0% in May, respectively. The hay used in this experiment averaged 12% crude protein and 58% total digestible nutrients (dry matter basis) over the 3-year period and compared to nutritive values reported for winter-annual pasture [14,15], it can be seen why this type of pasture complements warm-season grass hays and has been successfully used as a supplement for gestating beef cows [1-5,16]. Comparison of the nutritive value among cool-season annual grasses and clovers are few, but Lush [15] reported that the crude protein concentrations in pastures planted to a white clover and annual ryegrass mixture was normally 27% greater than monocultures of ryegrass. Further, Evans et al. [17] reported that white clover growing with perennial ryegrass increased the crude protein concentration of the grass by 9.2% because of atmospheric nitrogen fixation by *Rhizobium* bacteria. This increase in crude protein concentration of grasses grown in association with legumes has been documented for other species combinations [18-20]. Hence, pastures containing treatments WRR and WRW probably produced grasses that contained more crude protein than in pastures with the WRG treatment.

Conception rates for the cows on the WRG (73%) pastures tended to be less (*P* = 0.07) than the average of cows grazing WRR and WRW (84%). However, the conception rates between WRR and WRW did not differ (*P* ≥ 0.19). Conception rates from artificial insemination as calculated from difference in calving date and the artificial insemination breeding period did not differ (*P* ≥ 0.31) among pasture types (WRG = 16%, WRR = 20%, and WRW = 17%, SE = 0.06). Several experiments have shown that the *Bos indicus* species of cattle exhibit decreased reproductive function as day length is decreasing [21] and that interval from calving to initiation of cyclicity tends to be longer than in *Bos taurus* type cattle [22]. Additionally, *Bos indicus* cattle have shown increased anestrus during unfavorable breeding seasons [23]. Experiments have also shown that estradiol cypionate can have more variable results in insemination protocols than other estrogens with a longer half-life [24]. Hence, these factors more than likely contribute to the lower conception rates during the artificial insemination period. Conception rates during natural service also did not differ (*P* ≥ 0.29) among pasture types (WRG = 57%, WRR = 63%, and WRW = 68%, SE = 0.06), as well as post-partum intervals (*P* = 0.79, Table 1). Other research examining the use of cool-season annual grasses in the southern United States as a supplement for lactating beef cows during the winter has shown similar success at maintaining body weight and condition score, post-partum interval, and conception rates [2,3,5,16,25].

Calf body weight in January through March and at weaning (June) did not differ (*P* ≥ 0.21) between calves nursing cows grazing WRG and the average of calves nursing cows grazing WRR and WRW (Table 2). In April and May, calf body weight tended (*P* = 0.06) to be heavier for cattle grazing WRR and WRW pastures than for calves grazing WRG, but this trend diminished by weaning. Also, the body weight of calves nursing cows grazing WRR did not differ (*P* ≥ 0.70) from calves nursing cows grazing WRW. Average daily gain did not differ (*P* ≥ 0.37) between calves nursing cows grazing WRG and the average of calves nursing cows grazing WRR and WRW in any period (Table 2). Further, the average daily gain of calves nursing cows grazing WRR did not differ (*P* ≥ 0.41) from calves nursing cows grazing WRW. In southern Alabama, calf average daily gain of 0.89 kg was higher on rye/arrowleaf clover mixture than on a monoculture of annual ryegrass overseeded into bermudagrass [26].

**Table 2:** Birth weight, body weight, average daily gain, and weaning weight per cow exposed by calves nursing mature beef cows fed bermudagrass/bahiagrass hay supplemented by grazing on wheat/ryegrass pasture or wheat/ryegrass plus clovers over a 3-year period

**Item**	**Treatments**^a^			***P*-value**^b^		
	**WRG**	**WRR**	**WRW**	**SE**	**WRG versus Clovers**	**WRR versus WRW**
Birth weight, kg	34	36	35	0.9	0.17	0.89
Calf body weight, kg						
January	62	66	67	9.6	0.41	0.91
February	84	91	91	10.9	0.23	0.94
March	99	107	106	11.1	0.21	0.93
April	116	123	128	11.7	0.06	0.58
May	227	237	239	4.3	0.06	0.70
June (weaning)	244	254	253	6.7	0.29	0.88
Average daily gain, kg						
Birth to the start of grazing	0.92	0.92	0.95	0.086	0.71	0.41
January to weaning	0.98	1.02	0.99	0.035	0.37	0.44
Birth to weaning	0.93	0.96	0.95	0.023	0.39	0.57
Weaning weight/cow exposed, kg	173	212	209	12.0	0.02	0.86

^a^ WRG = winter-annual pasture composed of wheat and ryegrass; WRR = winter-annual pasture composed of wheat and ryegrass plus red clover, and WRW = winter-annual pasture composed of wheat and ryegrass plus white and crimson clover.

^b^ Contrasts: WRG vs Clover = WRG versus the average of WRR and WRW.

Because of multiplying effects of conception rate by cows to weaning weight gain by their nursing calves, calf weaning weight per cow exposed was 38 kg greater (*P* = 0.02) for cows collectively grazing WRR and WRW pastures than for cows grazing the WRG. This advantage means that cows grazing pastures with clovers produced 4.8 kg of calf body weight/kg of fertilizer nitrogen applied to the pasture, where the cows grazing only grass produced only 1.0 kg of calf body weight/kg of fertilizer nitrogen. Other research also demonstrated that overseeding with clovers alone with no nitrogen fertilizer resulted in calf body weight gains equal to those for annual ryegrass overseeded into bermudagrass and annually fertilized with 168 kg/ha N [26]. Reported nitrous oxide emission from pastures planted to mixtures of perennial grass and clovers in temperate zones are between 6 and 11 kg of nitrous oxide-nitrogen/ha annually [27,28]. Unfertilized temperate pastures receive the majority of their nitrogen supply for forage production from precipitation, mineralization of soil nitrogen, and fixation of atmospheric nitrogen [29]. Unfertilized perennial ryegrass pastures emitted only 6 kg of nitrogen/ha annually, whereas pastures fertilized with 200 kg of nitrogen/ha annually in the form of urea emitted 13 kg [7]. Hence, using a mixture of clovers and grasses for winter pasture should prove useful in maintaining production, reducing the need for nitrogen fertilization, to a small degree reducing nitrous oxide emission [30], and decreasing a system’s claim on fossil energy reserves [31].

## Conclusions

The results of this experiment show that by adding *Trifolium* clover species to winter-annual pasture mixtures for overseeding warm-season pastures can effectively supplement a beef cow herd, improve weaning weight per cow exposed, reduce the fertilizer nitrogen requirements needed to maintained sufficient production, and should result in decreased nitrous oxide emission from the pasture.

## Abbreviations

WRG, Winter-annual pasture composed of wheat and ryegrass; WRR, Winter-annual pasture composed of wheat and ryegrass plus red clover; WRW, Winter-annual pasture composed of wheat and ryegrass plus white and crimson clover.

## Competing interests

The authors declare no competing interest regarding the content or conclusions expressed in this research. Mention of trade names or commercial products in this article is solely for the purpose of providing specific information and does not imply recommendation or endorsement by the United States Department of Agriculture. All programs and services of the United States Department of Agriculture are offered on a nondiscriminatory basis without regard to race, color, national origin, religion, sex, age, marital status, or handicap.

## Authors’ contributions

SAG and PAB conceived the experiment and drafted the manuscript. WAW and TGM participated in the design and execution of the experiment. SAG and PAB preformed the statistical analysis. All authors have read and approved this manuscript.
